# Pigmentary Retinopathy in Alagille Syndrome: Fundus Findings in a Two-Year-Old Boy

**DOI:** 10.3390/diagnostics16020241

**Published:** 2026-01-12

**Authors:** Bogumiła Wójcik-Niklewska, Zofia Oliwa, Karina Dzięcioł, Adrian Smędowski

**Affiliations:** 1 Department of Pediatric Ophthalmology, Faculty of Medical Sciences in Katowice, Medical University of Silesia, 40-055 Katowice, Poland; asmedowski@sum.edu.pl; 2 Professor Kornel Gibiński University Hospital Center, Medical University of Silesia, 40-514 Katowice, Poland; 3 Students’ Scientific Society, Department of Ophthalmology, Faculty of Medical Sciences in Katowice, Medical University of Silesia, 40-752 Katowice, Poland; s86059@365.sum.edu.pl (Z.O.); s85696@365.sum.edu.pl (K.D.); 4 GlaucoTech Co., 40-282 Katowice, Poland

**Keywords:** Alagille syndrome, pigmentary retinopathy, optic disc anomalies, fundus photography, pediatric ophthalmology

## Abstract

Alagille syndrome (ALGS) is a rare autosomal dominant multisystem disorder characterized by bile duct paucity, congenital heart defects, characteristic facial features, skeletal anomalies, and distinctive ocular findings. Although anterior segment anomalies such as posterior embryotoxon are well recognized, posterior segment involvement has recently gained attention. We present fundus findings in a 2-year-old boy with genetically confirmed Alagille syndrome. Under general anesthesia, fundus examination revealed pink optic discs with blurred margins and drusen-like deposits, absence of the foveal reflex, and mottled hypopigmented and hyperpigmented areas that were consistent with retinal pigment epithelium (RPE) degeneration. Peripheral pigment clumping and RPE atrophy were also observed, while retinal vessels appeared normal. These features are characteristic of pigmentary retinopathy associated with ALGS and highlight the expanding spectrum of posterior segment changes in this condition. Recognition of such findings is essential, as they may contribute to visual impairment and support the systemic diagnosis.

**Figure 1:**
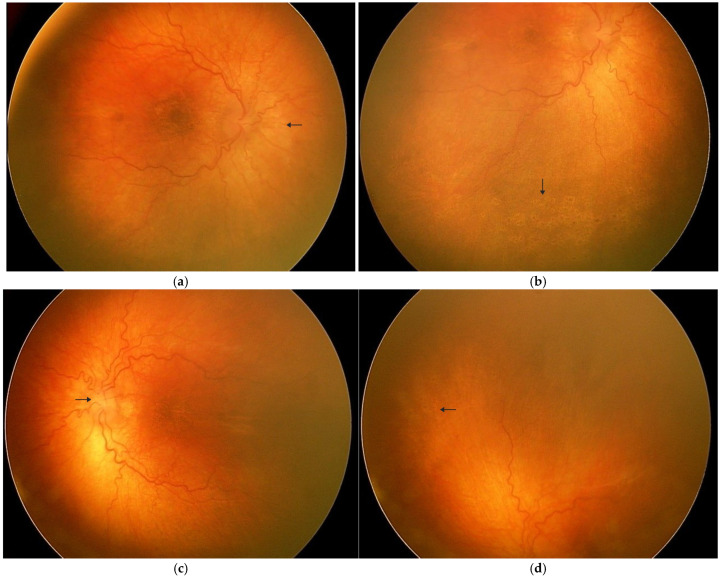
(**a**–**d**) We present a 2-year-old boy diagnosed with Alagille syndrome. On initial ophthalmic examination, both eyes demonstrated normal fixation and following movements, with appropriate pupillary light reactions. The intraocular pressure was measured at 19 mmHg in each eye. Cycloplegic refraction revealed +2.5 D sphere in the right eye and +3.0 D sphere in the left eye. Panels (**a**,**b**) show the right eye. In panel (**a**), the arrow indicates the optic disc with blurred margins, while in panel (**b**) the arrow highlights pigmentary changes in the posterior pole. Panels (**c**,**d**) show the left eye, demonstrating similar, largely symmetric findings. In panel (**c**), the arrow indicates the optic disc, whereas in panel (**d**), the arrow highlights pigmentary alterations in the peripheral retina. Fundus examination revealed bilateral, largely symmetric abnormalities. In both eyes, the optic discs appeared pink with slightly blurred margins and drusen-like deposits at the disc borders. The macular reflex was absent, with mottled areas of hypopigmentation and hyperpigmentation consistent with retinal pigment epithelium (RPE) degeneration. In the peripheral retina, clusters of pigment deposits alternated with patches of RPE atrophy, forming a pattern typical of pigmentary retinopathy. Retinal vessels were slightly tortuous, but their caliber was preserved, and no marked vessel attenuation, bone-spicule pigmentation, or inflammatory scars were observed. These findings are consistent with pigmentary retinopathy typically described in patients with Alagille syndrome. The diagnosis of Alagille syndrome was established based on characteristic clinical features, including chronic cholestasis, typical facial dysmorphism, congenital heart disease, and skeletal anomalies, in accordance with established diagnostic criteria. The patient was evaluated by a clinical geneticist; however, formal documentation of molecular testing was not available for inclusion in this report. Alagille syndrome (ALGS) is a rare autosomal dominant multisystem disorder most commonly caused by pathogenic variants in JAG1 and, less frequently, NOTCH2. The condition is classically defined by five major clinical features: chronic cholestasis due to intrahepatic bile duct paucity, characteristic facial dysmorphisms, congenital cardiac defects, skeletal anomalies (particularly butterfly vertebrae), and ocular findings [1]. However, clinical expression is highly variable, and ocular abnormalities may be among the most consistent diagnostic clues. The most characteristic ophthalmic feature in ALGS is posterior embryotoxon, reported in up to 95% of affected individuals [1,2]. Other anterior segment anomalies include Axenfeld anomaly and angle abnormalities, which may predispose to secondary glaucomatous changes [1]. In addition to anterior segment involvement, posterior segment findings have increasingly been recognized. Optic disc anomalies, such as optic pits and colobomas, have been described [1,3], along with vascular tortuosity and abnormal branching patterns of the retinal vasculature [1,2]. More recently, macular involvement has been highlighted as a significant manifestation, with cases of macular atrophy and disruption of foveal architecture particularly associated with JAG1-related disease [4,5]. Furthermore, a spectrum of pigmentary retinopathy ranging from subtle mottling to severe retinitis pigmentosa-like changes has been reported, which may contribute to progressive visual dysfunction [2,5]. From a differential diagnostic perspective, pigmentary retinopathy in early childhood may arise from a number of conditions, and these should be briefly considered to contextualize the present case. Inherited retinal dystrophies, including retinitis pigmentosa and syndromic ciliopathies, classically show progressive vessel attenuation, bone-spicule pigmentation, nyctalopia, and gradual peripheral visual field loss [6]. In contrast, our patient demonstrated preserved vessel caliber, absence of bone-spicule-type changes, and no symptoms suggestive of night blindness. Moreover, the very early presentation, in the setting of a multisystem disorder with a defined genetic background, makes a primary photoreceptor dystrophy less likely. Inflammatory or post-infectious etiologies, such as TORCH-related chorioretinitis, typically leave chorioretinal scars, pigment clumping surrounding healed lesions, or vitreous inflammatory cells [7]. These signs were not observed in our case, and there was no relevant perinatal or infectious history. Autoimmune retinopathies, although rare in children, are usually characterized by rapid visual decline and may show associated inflammatory features, both of which were absent [8]. These considerations support the interpretation that the pigmentary changes represent part of the posterior segment spectrum of Alagille syndrome rather than an alternative hereditary, inflammatory, or autoimmune process. The pathophysiology of posterior segment involvement in Alagille syndrome remains uncertain. Because JAG1/NOTCH2 mutations disrupt the Notch signaling pathway, which is essential for retinal and vascular development, abnormal signaling during ocular maturation may plausibly contribute to the pigmentary changes and optic disc anomalies observed in affected patients [9]. Experimental studies support an effect of altered Notch signaling on retinal architecture; however, a direct mechanistic link specific to ALGS has not yet been demonstrated. Although pigmentary retinopathy has been described in Alagille syndrome, most published reports involve older children or adolescents, and detailed documentation in very young patients remains limited. Our case demonstrates posterior segment involvement at only 2 years of age, with bilateral and largely symmetric pigmentary changes coexisting with blurred optic disc margins and drusen-like deposits. This finding supports the concept that retinal pathology in ALGS may emerge early in life, even in the absence of advanced functional impairment, and highlights the importance of systematic ophthalmic surveillance in affected children. The photographic documentation further contributes to the growing recognition of pigmentary retinopathy as part of the ocular spectrum of Alagille syndrome. Taken together, the breadth of ocular manifestations in ALGS underscores the importance of a comprehensive ophthalmic examination as part of the multidisciplinary evaluation. Recognition of these findings not only supports the systemic diagnosis but also facilitates appropriate long-term monitoring, as certain features may be progressive and impact visual prognosis. Limitations: A limitation of this report is the absence of OCT and other advanced imaging modalities, which might have provided additional structural information. However, obtaining such imaging was not feasible in this very young child outside of general anesthesia.

## Data Availability

No new data were created or analyzed in this study. Data sharing is not applicable to this article.
